# Maternal obesity in low-resource settings: a multicenter cohort study of labor and neonatal outcomes in Guinea

**DOI:** 10.3389/fmed.2025.1584650

**Published:** 2025-11-20

**Authors:** Abdourahamane Diallo, Elhadj Mamoudou Bah, Ibrahima Koussy Bah, Telly Sy, Lothaire Ayadjenou Hounga, Fiona Corbaz, David Desseauve

**Affiliations:** 1 Gynecology and Obstetrics Unit of National Ignace Deen Hospital, Conakry, Guinea; 2 Health Science and Technic Faculty of Gamal Abdel Nasser University of Conakry, Conakry, Guinea; 3 Gynecology and Obstetrics Unit of National Donka Hospital, Conakry, Guinea; 4 Obstetric Unit, Department Woman-Mother-Child, Lausanne University Hospital, Lausanne, Switzerland; 5 Obstetric Units, Child Couple University Hospital, University of Grenoble, Grenoble, France

**Keywords:** obesity, pregnancy outcome, obstetrical outcome, labor, childbirth, perinatal care, Sub-Saharan Africa, Western Africa

## Abstract

**Objective:**

This study aimed to evaluate the impact of maternal obesity on obstetrical outcomes, including labor and delivery parameters, as well as maternal and neonatal prognosis.

**Study design:**

This observational multicenter cohort study was conducted over 6 months in Conakry, where Guinea’s two busiest maternity hospitals are located. A total of 295 obese women (body mass index (BMI) > 30 kg/m2) and 590 normal-weight women (BMI 18.5–24.9 kg/m2) were included. Obstetrical characteristics and outcomes were compared between obese and normal-weight parturients.

**Results:**

Compared to normal-weight women, obese parturients had significantly higher risks of labor induction (RR = 1.6, 95% CI [1.1–2.3]), occipital-posterior fetal position (RR = 1.8, 95% CI [1.3–2.8]), prolonged second stage of labor (RR = 1.7, 95% CI [1.2–2.3]), and oxytocin administration for uterine hypo-contractility (RR = 1.8, 95% CI [1.3–2.4]). Increased rates were also observed for episiotomy (RR = 2.5, 95% CI [1.6–3.9]), vacuum-assisted delivery (RR = 1.9, 95% CI [1.1–3.6]), cesarean section (RR = 1.7, 95% CI [1.3–4.4]), postpartum hemorrhage (RR = 1.8, 95% CI [1.3–5.2]), and postcesarean wound infection (RR = 3.3, 95% CI [2.2–19.6]). Neonates born to obese women were at an increased risk of perinatal asphyxia (RR = 2.9, 95% CI [1.3–6.4]), low APGAR score both at 1 min (RR = 1.7, 95% CI [1.3–2.2]) and 10 min (RR = 1.7, 95% CI [1.2–2.5]), and the need for neonatal resuscitation (RR = 1.6, 95% CI [1.2–2.1]). No significant differences were observed between groups regarding the risk of breech presentation, the type of cephalic presentation (occipital-anterior versus occipital-posterior), or neonatal mortality.

**Conclusion:**

In low-income settings, maternal obesity is associated with a significantly increased risk of adverse labor, delivery, and perinatal outcomes—mirroring patterns observed in higher-resource contexts. These findings underscore the need for enhanced healthcare provider training and the implementation of targeted maternal weight management strategies. Moreover, obstetrical protocols and clinical guidelines should be adapted based on maternal BMI to better address the specific risks associated with obesity in pregnancy.

## Introduction

The global prevalence of overweight and obesity is increasing at an alarming rate, representing a major public health crisis affecting both high- and low-resource countries alike (1). In low-income settings, the availability and reliability of data concerning obstetrical outcomes are often limited, highlighting the urgent need to strengthen research efforts in these contexts.

A large-scale analysis of overweight and obesity among women across 32 Sub-Saharan African countries revealed that excess weight also poses a substantial burden in this region, with an overall prevalence of 15.9% (2). A 2015 meta-analysis estimated that the prevalence of obesity among women in their first trimester of pregnancy in Africa could reach 17.9% (3). Similarly, a retrospective cohort study from Northern Tanzania reported that, between 2000 and 2015, 26.5% of pregnant women were overweight and 12.3% had obesity during their first trimester (4).

Maternal obesity is a well-established risk factor for numerous maternal and fetal complications occurring throughout pregnancy, labor, and the postpartum period (5, 6). The onset of labor involves complex physiological processes, including functional progesterone withdrawal, increased estrogenic activity, upregulation of contraction-associated proteins (e.g., oxytocin and prostaglandin receptors), the activation of pro-inflammatory cytokines, and the transition of the myometrium to a contractile phenotype (7, 8). In women with obesity, these processes are disrupted due to delayed cervical ripening, reduced sensitivity to prostaglandins, diminished expression of myometrial oxytocin receptor and gap junction proteins, dysregulated adipokine and cytokine signaling, and increased lipid deposition in the myometrium. These alterations result in impaired uterine contractility and increased rates of labor dysfunction when compared to women of normal weight (7–9).

In this population, the onset of labor is frequently delayed (6, 8), leading to a greater reliance on labor induction techniques (4, 6, 8). Paradoxically, maternal overweight and obesity are also linked to an increased risk of preterm labor (6, 8). Furthermore, women with higher body mass index (BMI) are more likely to undergo cesarean delivery compared to their normal-weight counterparts (6, 10). The postpartum period is similarly affected, with higher incidences of complications such as postpartum hemorrhage (4, 6) and infection (6, 8, 11). In terms of neonatal outcomes, infants born to obese mothers are at an elevated risk for macrosomia, admission to neonatal intensive care units, congenital anomalies, and stillbirth (6, 8, 12–14).

Despite the well-documented risks associated with maternal obesity in high-resource settings, its impact in low-income countries remains poorly characterized, particularly where healthcare resources are limited, and complications may be further exacerbated. In light of this gap, we conducted a bicentric comparative study to evaluate the effect of maternal obesity on obstetrical outcomes in Guinea, a low-income country. We hypothesized that maternal obesity is associated with a greater risk of adverse obstetrical outcomes when compared to non-obese women.

## Methods

### Study population and study design

This multicenter, observational, and analytical cohort study was conducted over 6 months, from 1 July to 31 December 2021. The research was conducted in two maternity units in Conakry, Guinea: the National Ignace Deen Hospital in Conakry, a tertiary-level referral center, and the Center Médico-Communal in Ratoma, a secondary-level facility. These institutions report approximately 6,500 and 3,000 deliveries per year, respectively, and were selected based on their high patient volumes.

All pregnant women presenting for their final prenatal consultation at either of the two study sites during the data collection period were assessed for eligibility according to predefined inclusion and exclusion criteria. Eligible participants were women with a singleton, physiological pregnancy beyond 37 weeks of gestation, determined either by the first day of the last menstrual period or ultrasonographic dating, and a BMI falling within either the obese range (BMI ≥ 30 kg/m^2^) or the normal range (BMI 18.5–24.9 kg/m^2^), as defined by the World Health Organization (WHO). Informed consent was required, and participants had the intent to deliver at one of the two study centers.

The exclusion criteria comprised any condition likely to interfere with the normal course of labor, including uterine abnormalities (e.g., fibroid, malformed, or scarred uterus), pelvic abnormalities, multiple gestations, fetal malformations, or intrauterine fetal death diagnosed before the onset of labor.

### Data source and data set

Data for this study were obtained through structured patient interviews, maternal clinical examinations, and standardized neonatal assessments using a dedicated data collection form developed explicitly for this study. Data were collected during labor and the immediate postpartum period—the first 6 h following delivery—or where complications arose until maternal or neonatal stabilization was achieved.

The data collection team consisted of trained obstetricians, midwives, and pediatricians. The standardized forms captured comprehensive information on sociodemographic characteristics, obstetric variables, and maternal and neonatal outcomes. Sociodemographic data included maternal age, marital status, educational level, and occupation. Obstetric parameters encompassed the mode of labor onset (spontaneous or induced), fetal presentation, cephalic presentation type at delivery (occiput-anterior or occiput-posterior), duration of the second stage of labor (with a cutoff of 30 min, as no epidural is available), use of oxytocin infusion, the performance of episiotomy, vacuum-assisted delivery, cesarean section, postpartum hemorrhage, postcesarean wound infection, and maternal mortality. Neonatal outcomes included the APGAR score at 1 and 10 min, the need for neonatal resuscitation, and neonatal mortality. According to American College of Obstetricians and Gynecologists (ACOG) guidelines (15), an APGAR score above 7 was considered reassuring, while a score below 7 was classified as abnormal.

### Statistical analysis

Data analysis was performed using Epi Info version 7 (Centers for Disease Control and Prevention, Atlanta, GA, USA). Descriptive statistics were applied to summarize the data: proportions were used for qualitative variables, and means, standard deviations, and range (minimum and maximum values) were reported for quantitative variables. Categorical variables were compared using Pearson’s chi-squared test. The impact of maternal obesity on obstetrical outcomes was evaluated by calculating relative risks (RR) and their corresponding 95% confidence intervals (CI). A *p*-value of less than 0.05 was considered indicative of statistical significance.

### Compliance with ethical standards

In accordance with ethical requirements for observational studies, authorization to conduct this research was obtained both orally from the heads of the two participating maternity units—National Ignace Deen Hospital in Conakry and the Center Médico-Communal in Ratom—and in writing from the local ethics committee, the Commission d’Éthique pour la Recherche de la Chaire de Gynécologie-Obstétrique, before the initiation of the study (Approval number: 0112/CME/FSTS/UGANC/2021).

The research team provided all participants a comprehensive verbal explanation of the study’s objectives, procedures, and data confidentiality protocols by reading an informed consent statement aloud. Participation was entirely voluntary, and oral consent was obtained from each participant. For minor participants, oral consent was required from both the participant and her spouse; parental consent was also obtained if one or both parents were present.

All individual-level data were collected solely for scientific purposes and were processed in accordance with national data protection regulations in Guinea. Strict confidentiality protocols were implemented to ensure the privacy and protection of all participants throughout the study.

## Results

### Characteristics of the study population

**Figure 1:**
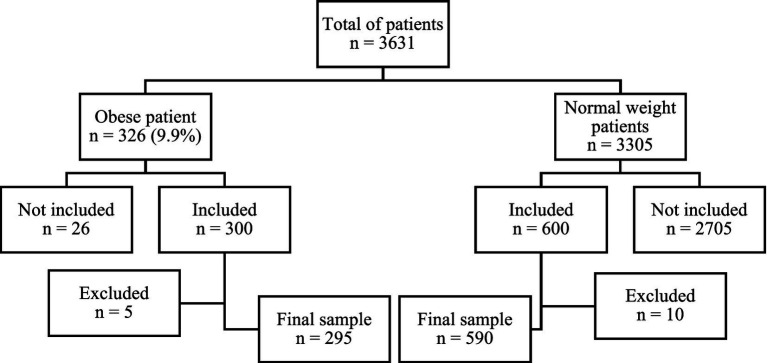
Flowchart of the study population.

A total of 3,631 pregnant women attended the final prenatal consultation at the two participating maternity centers during the study period. Among them, 326 women (9.9%) met the criteria for obesity. Following the application of exclusion criteria, 295 obese women were retained for analysis, while 31 were excluded due to non-eligibility or loss of follow-up (Figure 1).

The mean age of the obese participants was 30.6 years, with the majority (65.4%) aged between 25 and 34 years. Most obese women were married (92.5%), and nearly half (47.5%) had completed higher education. A substantial proportion (66.7%) were professionally active as government employees or independent workers.

In comparison, the mean age in the normal-weight group was slightly higher, at 31.2 years. Although the proportion of married women remained high in this group (85.1%, *p* < 0.05), a significantly larger percentage had never attended school (34.7%, *p* < 0.05). Additionally, the majority of normal-weight women were housewives (68%), whereas only 18.8% were reported to be salaried or self-employed workers (*p* < 0.05) (Table 1).

**Table 1:** Sociodemographic characteristics of the study population.

Characteristics	Obese *n* (%)	Normal weight *n* (%)	*p*-value
Age [years]			> 0.6
15–24	31 (10.5)	65 (11.0)	
25–34	193 (65.4)	377 (63.9)	
≥ 35	71 (24.1)	148 (25.1)	
Mean	30.6 ± 5.3	31.2 ± 5.1	
Marital status			< 0.001
Married	273 (92.5)	502 (85.1)	
Single	18 (6.1)	38 (6.4)	
Widow	1 (0.3)	20 (3.4)	
Divorced	3 (1.0)	30 (5.1)	
Level of education			< 0.001
Non-educated	98 (33.2)	205 (34.7)	
Primary school	24 (8.1)	187 (31.7)	
Secondary school	33 (11.2)	122 (20.7)	
Superior education	140 (47.5)	76 (12.9)	
Profession			< 0.001
Housewife	58 (19.7)	401 (68.0)	
Student	40 (13.6)	78 (13.2)	
Freelancer	102 (34.5)	70 (11.9)	
State employee	95 (32.2)	41 (6.9)	

### Labor and delivery impacts of maternal obesity

Our findings demonstrate a 60% increased risk of labor induction among obese women (RR = 1.6, 95% CI [1.1–2.3]). No significant association was observed between obesity and cephalic or breech presentation risk. However, the prevalence of occiput-posterior presentation was significantly higher in the obese group (RR = 1.8, 95% CI [1.3–2.8]).

Obese women also experienced a prolonged second stage of labor, particularly those with fewer than four previous deliveries. Furthermore, obesity was significantly associated with a higher need for oxytocin administration, a twofold increase in the rate of episiotomy, and nearly twice the frequency of operative vaginal delivery by vacuum extraction. A strong association was observed between obesity and cesarean delivery (RR = 1.7, 95% CI [1.3–4.4]) (Table 2).

**Table 2:** Comparison of obstetrical outcomes in obese and normal-weight parturients.

Obstetrical outcomes	Obese *n* (%)	Normal weight *n* (%)	Chi^2^	*p* value	RR [IC 95%]
Labor start mode	*n* = 295	*n* = 590			
Artificial	53 (18)	68 (11.5)	6.91	<0.01	1.6 [1.1–2.3]
Presentation mode	*n* = 295	*n* = 590			
Breech	22 (7.5)	31 (5.3)	1.7	>0.10	1.4 [0.8–2.3]
Type of cephalic	*n* = 273	*n* = 559			
Not vertex/occiput	9 (3.3)	21 (3.8)	0.1	>0.5	0.9 [0.7–1.1]
Orientation of the presentation at birth if vertex	*n* = 264	*n* = 538			
Occipital-posterior	56 (21.2)	64 (11.9)	12.1	<0.001	1.8 [1.3–2.8]
Occipital-posterior presentation at the extraction	*n* = 39	*n* = 55			
Occipital-anterior	27 (69.2)	49 (96.4)	5.7	<0.02	8.6 [1.5–48.1]
Duration of second stage of labor (min)	*n* = 218	*n* = 500			
>30 min, all parities	57 (26.1)	77 (15.4)	11.5	<0.001	1.7 [1.2–2.3]
>30 min, parity <4	34 (23.1)	52 (13.7)	6.9	<0.01	1.7 [1.2–2.5]
>30 min, parity ≥ 4	15 (21.1)	19 (15.7)	0.9	>0.3	1.3 [1.0–1.7]
Oxytocin perfusion	*n* = 295	*n* = 590			
Yes	64 (21.7)	72 (12.2)	13.7	<0.001	1.8 [1.3–2.4]
Episiotomy	*n* = 218	*n* = 500			
Yes	34 (15.6)	31 (6.2)	16.3	<0.001	2.5 [1.6–3.9]
Vacuum use	*n* = 218	*n* = 500			
Yes	16 (7.3)	19 (3.8)	4.2	<0.05	1.9 [1.1–3.6]
Cesarean section	*n* = 295	*n* = 590			
Yes	77 (26.1)	90 (15.3)	15.0	<0.001	1.7 [1.3–4.4]
Postpartum hemorrhage	*n* = 295	*n* = 590			
Yes	51 (17.3)	58 (9.8)	10.2	<0.01	1.8 [1.3–5.2]
Wound infection postcesarean section	*n* = 77	*n* = 90			
Yes	14 (18.2)	5 (5.6)	6.5	<0.05	3.3 [2.2–19.6]
Maternal death	*n* = 295	*n* = 590			
Yes	0 (0.0)	0 (0.0)			
Perinatal asphyxia	*n* = 295	*n* = 590			
Yes	13 (4.4)	9 (1.5)	6.9	<0.01	2.9 [1.3–6.4]
APGAR score at 1 min	*n* = 295	*n* = 590			
<7	74 (25.1)	89 (15.1)	13.1	<0.001	1.7 [1.3–2.2]
APGAR score at 10 min	*n* = 295	*n* = 590			
<10	44 (14.9)	51 (8.6)	8.2	<0.01	1.7 [1.2–2.5]
Neonatal resuscitation	*n* = 295	*n* = 590			
Yes	70 (23.7)	85 (14.4)	12.0	<0.001	1.6 [1.2–2.1]
Neonatal death	*n* = 295	*n* = 590			
Yes	14 (4.7)	27 (4.6)	0.1	>0.9	1.0 [1.0–1.0]

### Maternal prognosis and maternal obesity

Obese women were significantly more likely to experience postpartum hemorrhage (RR = 1.8, 95% CI [1.3–5.2]). Additionally, the risk of postcesarean wound infection was more than three times higher among obese women compared to their normal-weight counterparts (RR = 3.3, 95% CI [2.2–19.6]). Notably, no maternal deaths were recorded during the study period.

### Fetal prognosis and maternal obesity

Maternal obesity was significantly associated with an increased risk of perinatal asphyxia, defined by fetal heart rate abnormalities and the presence of meconium-stained amniotic fluid (RR = 2.9, 95% CI [1.3–6.4]), as detected by Pinard stethoscope or Doppler ultrasound.

Newborns of obese mothers were more likely to present with APGAR scores of ≤7 at both 1 and 10 min of life (RR = 1.7, 95% CI [1.3–2.2] and RR = 1.7, 95% CI [1.2–2.5], respectively). Although no significant association was found between maternal obesity and neonatal mortality, a strong correlation was observed with the need for neonatal resuscitation (*p* < 0.01, RR = 1.6, 95% CI [1.2–2.1]) (Table 2).

## Discussion

### The main findings of the study

In this multicenter analytic cohort conducted in a low-income setting, the prevalence of obesity was approximately 10%, notably lower than thr figures reported in other sub-Saharan African studies, which range from 12.3 to 17.9% in the first trimester of pregnancy (2–4). Genetic or nutritional factors may contribute, but the reasons for this discrepancy remain unclear.

Maternal obesity was associated with a significantly increased likelihood of labor induction—a 60% higher rate compared to normal-weight women. This finding, consistent with prior literature (4, 6, 8), reflects common clinical efforts to mitigate the risk of stillbirth in obese pregnancies. Obesity emerged as an independent risk factor for labor induction, likely due to a confluence of factors, including delayed spontaneous labor onset, hypertensive disorders, gestational diabetes, and fetal macrosomia (4, 6, 8, 13–15).

Although no significant association was found between maternal obesity and non-cephalic presentation, a higher prevalence of occiput-posterior positioning was observed among obese women with vertex presentations. This finding is supported by Cheng et al. (16) and may be attributed to fat deposition in the maternal pelvis, leading to soft tissue dystocia (17). Furthermore, excessive gestational weight gain and macrosomia may hinder fetal rotation during labor, as previously suggested (18).

Among non-multiparous obese women (i.e., those with fewer than four previous deliveries, as defined in the Sub-Saharan African context (19)), the second stage of labor—which corresponds to the period from full cervical dilation until delivery of the baby—was more likely to be prolonged (RR: 1.7). While the literature remains divided on this topic, the majority of studies confirm prolonged second stage of labor in obese women, although findings on the exact duration vary (13, 20, 21).

A higher rate of oxytocin administration was observed in obese patients, plausibly due to impaired uterine contractility. Zhang et al. (20) attribute this to diminished calcium influx in the myometrium, while Carlson et al. further implicate delayed cervical ripening, altered prostaglandin sensitivity, amniotic membrane strengthening, reduced oxytocin receptor expression in the myometrium, and impaired myocyte contractility and gap junction formation (8).

We also found a significantly increased likelihood of episiotomy among obese parturients. This contradicts previous findings (3) and may reflect a clinical response to the higher rate of occiput-posterior presentations.

Vacuum-assisted deliveries were nearly twice as frequent in obese women, a finding that diverges from some reports in the literature and may reflect institutional practice or clinician training (22). Cesarean delivery rates were significantly elevated among obese patients, aligning with previous studies (6, 23). However, some research suggests that BMI may not independently predict cesarean risk in the population of African descent (24).

Postoperative wound infections were three times more frequent in obese women, echoing observations by Wloch et al. (25). Contributing factors likely include suboptimal antibiotic dosing and poor adipose tissue perfusion, impairing tissue healing (25).

Obese women were also at an increased risk of postpartum hemorrhage, likely due to uterine atony, extended operative times, and macrosomia (3, 6).

Neonates born to obese mothers showed significantly higher rates of perinatal asphyxia and lower APGAR scores at both 1 and 10 min of life, often requiring resuscitation. These findings are well-documented and likely reflect an increased risk of gestational complications, such as gestational diabetes and pre-eclampsia, in obese women, leading to worse neonatal outcomes (26). However, our study found no significant difference in neonatal mortality between obese and normal-weight groups. This discrepancy—between poor immediate neonatal outcomes and preserved survival—may reflect enhanced surveillance and clinical responsiveness within the study cohort.

### Implications for the field

These findings underscore the urgent need to address maternal obesity as a public health priority, particularly in low-resource settings, where the capacity to manage obstetric complications is limited. The amplified impact of obesity on labor outcomes in such environments calls for targeted interventions, including pre-conception counseling for at-risk women, nutritional and weight management programs during pregnancy, the development of context-specific clinical protocols for the management of obese parturients, and enhanced provider training in managing labor complications related to obesity. These strategies could inform national prenatal care guidelines, strengthen obstetric services, and guide policymaking to reduce preventable maternal and neonatal morbidity.

### Strengths and limitations of the study

A major strength of this study lies in its multicentric, analytic cohort design, which allows for generalizability across similar low-resource contexts. The study also benefits from robust statistical power to assess the impact of obesity on the delivery process.

However, several limitations warrant consideration. First, we lacked follow-up data beyond the immediate postpartum period, limiting our ability to assess delayed maternal or neonatal complications.

A second limitation of our study is the absence of overweight women (BMI 25.0–29.9 kg/m^2^) in the analytical cohort. The study was intentionally designed to compare two distinct BMI categories—normal weight and obesity—based on clear WHO definitions and established clinical thresholds. While this binary classification improves methodological clarity and statistical power, it does not capture potential gradations of risk across the full BMI continuum. In particular, the overweight population may present intermediate or mixed risk profiles that could contribute valuable insights. Similarly, we were not able to further stratify the obese cohort into obesity classes I, II, and III due to limited sample sizes in these subgroups. These aspects merit attention in future larger-scale or multicenter studies with sufficient statistical power to investigate dose–response relationships between increasing BMI and obstetric outcomes.

Third, key potential confounders—such as the presence and severity of gestational diabetes, hypertensive disorders, or differences in access to care—were not fully accounted for in the analysis. The absence of this information may have influenced the observed associations.

Fourth, information on parity and gravidity was not included in our analysis, although these are important variables that may significantly influence obstetric outcomes. Their absence represents another limitation of our study, and we suggest that future research on this topic incorporate these factors to better delineate their potential role.

Additionally, the finding of increased neonatal morbidity without a corresponding rise in mortality deserves further investigation, particularly concerning the role of enhanced intrapartum monitoring and neonatal resuscitation practices.

## Conclusion

In this multicenter study from Guinea, maternal obesity was strongly associated with adverse labor and delivery outcomes, including higher rates of labor induction, cesarean section, operative vaginal delivery, postpartum hemorrhage, and neonatal compromise. These associations persisted despite a lack of significant difference in neonatal mortality, likely due to close clinical monitoring.

Given the rising prevalence of obesity among pregnant women in sub-Saharan Africa, there is an urgent need to integrate targeted interventions—such as preconception counselling and weight management—into prenatal care programs. Policymakers and clinicians in similar low-income settings must adapt perinatal care protocols to anticipate better and manage the complex risks posed by maternal obesity. Future research should explore tailored clinical pathways and evaluate the long-term outcomes for mothers and neonates.

## Data Availability

The datasets presented in this article are not readily available because the data is not available. Requests to access the datasets should be directed to DDesseauve@chu-grenoble.fr.
